# Basic Exploratory Research versus Guideline-Compliant Studies Used for Hazard Evaluation and Risk Assessment: Bisphenol A as a Case Study

**DOI:** 10.1289/ehp.0900893

**Published:** 2009-06-29

**Authors:** Rochelle W. Tyl

**Affiliations:** RTI International, Research Triangle Park, North Carolina, USA

**Keywords:** basic/exploratory studies, bisphenol A, end points, 17β-estradiol, guideline-compliant studies, routes of administration

## Abstract

**Background:**

Myers et al. [Environ Health Perspect 117:309–315 (2009)] argued that Good Laboratory Practices (GLPs) cannot be used as a criterion for selecting data for risk assessment, using bisphenol A (BPA) as a case study. They did not discuss the role(s) of guideline-compliant studies versus basic/exploratory research studies, and they criticized both GLPs and guideline-compliant studies and their roles in formal hazard evaluation and risk assessment. They also specifically criticized our published guideline-compliant dietary studies on BPA in rats and mice and 17β-estradiol (E_2_) in mice.

**Objectives:**

As the study director/first author of the criticized E_2_ and BPA studies, I discuss the uses of basic research versus guideline-compliant studies, how testing guidelines are developed and revised, how new end points are validated, and the role of GLPs. I also provide an overview of the BPA guideline-compliant and exploratory research animal studies and describe BPA pharmacokinetics in rats and humans. I present responses to specific criticisms by Myers et al.

**Discussion and conclusions:**

Weight-of-evidence evaluations have consistently concluded that low-level BPA oral exposures do not adversely affect human developmental or reproductive health, and I encourage increased validation efforts for “new” end points for inclusion in guideline studies, as well as performance of robust long-term studies to follow early effects (observed in small exploratory studies) to any adverse consequences.

Differences in perspective on basic research studies versus large-scale, guideline-compliant studies evaluating the potential developmental/reproductive effects of bisphenol A (BPA) reported by Myers et al. (2009) reflect disagreement in and ignorance of the roles and strengths and weaknesses of these study types. This controversy affects BPA safety assessment, which has become a major public policy issue. In this article I review characteristics and uses of basic/exploratory research versus regulatory guideline-compliant studies, using BPA as a case study.

## Background

Basic/exploratory research in toxicology is conducted to identify unknown potential hazards, elucidate the mode/mechanism of action for known toxicants, or explore novel end points for possible subsequent formal validation. These studies commonly employ routes of administration and/or doses that are not relevant for humans; include few dose groups and few animals per group, and/or nonvalidated end points; are not tracked to adverse outcomes; and are typically creative, short term, relatively inexpensive, and funded by universities, government grants, and nongovernmental organizations (NGOs). These basic research studies play significant roles but are limited in assessing potential risks to human health. To assess risks, governmental agencies rely on guideline-compliant studies using appropriate exposures (routes, doses, durations), validated end points linked to adverse outcomes, and appropriate group sizes and numbers. Exploratory research and risk assessment studies are interactive and iterative (National Research Council 2000).

Risk-relevant studies are performed under regulatory testing guideline (TG) protocols and Good Laboratory Practices (GLPs). The Food and Drug Administration (FDA 1978) established standards for design, conduct, and reporting of nonclinical laboratory studies in support of applications for FDA marketing permits. The U.S. Environmental Protection Agency (EPA) prescribed GLP standards for studies on health and environmental effects and chemical fate testing under the Federal Insecticide, Fungicide and Rodenticide Act (FIFRA; U.S. EPA 1983) and under the Toxic Substances Control Act (TSCA; U.S. EPA 1989). The Organisation for Economic Co-operation and Development (OECD) has also established GLPs (OECD 1998). The U.S. EPA may consider data not developed in accordance with these provisions “insufficient to evaluate the health effects, environmental effects, and fate of the chemical.” (U.S. EPA 1985).

Guideline-compliant studies evaluate potential hazard and risk of substances and are performed following/exceeding governmental TGs and GLPs. Guideline-compliant multi-generational reproductive toxicity studies, with large numbers of animals per group per generation, are very expensive and typically funded by manufacturers, consortia of manufacturers, and/or governments. These studies are necessary for hazard evaluation and/or risk assessment because of their statistical power to detect reproducible effects linked to adverse outcomes; relevant exposure routes, doses, and animal models; and dose–response assessment. GLPs require complete, permanent documentation of staff; valid study design; standard operating procedures (SOPs); training, performance, formulation, and statistical analyses; and retention of summary/individual data, so there is confidence in the study design, performance, and results, and anyone can subsequently fully reconstruct the study.

Harmonization of TGs by the OECD [United States (U.S. EPA), Japan, and Europe] for industrial chemicals, and by the International Conference on Harmonization (European Commission, Japanese Ministry, U.S. FDA, and pharmaceutical manufacturers) for pharmaceuticals, ensures guidelines are globally consistent and acceptable. Development and revision of governmental regulatory guidelines involve experts from government, academia, and industry evaluating the strengths and weaknesses of the current and proposed TGs and end points. For the revised two-generation study TGs, new validated end points include anogenital distance (AGD), retained nipples/areolae, acquisition of puberty, estrous cyclicity, andrology, and ovarian primordial follicle counts. Proposed guidelines (and/or new end points) are published for comment (e.g., U.S. EPA 1996). After comments and revisions, the final guideline is published [U.S. EPA TSCA new two-generation guideline (U.S. EPA 1997); U.S. EPA Office of Prevention, Pesticides, and Toxic Substances (OPPTS; including TSCA and FIFRA) version 1998; and the new OECD version (OECD 2001)]. TGs evolve over time, and appropriate changes are incorporated as described above. If/when researchers identify new end points for inclusion, there is a defined process to follow. Virtually all new validated end points in current guidelines originated in exploratory research.

When a government guideline is developed for a “new” study type, such as the rat ovariectomized uterotrophic and castrate Hershberger assays for endocrine disruptors, a laboratory evaluates the protocol’s variables, and governmental, industrial, and contract testing laboratories run the protocol with known (and coded) chemicals. A background review document with laboratory results and a draft guideline are published. The process involves scientists, testing laboratories, governmental oversight, and peer review before the guideline and/or end point(s) is officially acceptable. Although TGs are comprehensive and provide a robust baseline standard, one can also add evaluations to enhance the ability of the guideline to detect adverse effects.

Guideline studies are required for governmental risk assessment and critical for risk evaluations by other agencies, such as the National Toxicology Program (NTP 2008), the Center for the Evaluation of Risks to Human Reproduction (CERHR 2008), and the European Food Safety Authority (EFSA 2006). Guideline studies can inform further exploratory research and are universally considered robust, reliable, and essential to confirm, refute, and/or expand exploratory studies. When appropriate, nonvalidated end points can be used in formal risk assessments as “points of departure,” but only when the end point is known to relate directly to an adverse outcome; for example, fetal testicular testosterone content was used as the point of departure for a U.S. EPA phthalate risk assessment because the direct adverse consequences from low fetal testosterone were known.

BPA is a high-production-volume chemical. Ubiquitous human exposure to very low BPA doses occurs primarily through consumption of food that comes in contact with materials containing BPA, such as polycarbonate plastics and epoxy resins (manufactured from BPA) used in plastic bottles, and food/beverage container coatings (EFSA 2006). There is considerable debate on whether low oral BPA doses cause reproductive/developmental effects in animals. Guideline-compliant BPA studies have shown effects only at high doses, which also caused parental systemic toxicity, but have not reproduced the low-dose effects reported in exploratory BPA studies.

Myers et al. (2009) criticized GLP-compliant studies for serious conceptual and methodologic flaws, failure to address replication, and using methods not sensitive or “state of the art” and opined that these studies cannot detect effects of low-dose BPA. They specifically criticized our BPA [and 17β-estradiol (E_2_)] dietary studies (Tyl et al. 2002, 2008a, 2008b, 2008c).

## Guideline-Compliant BPA Studies

Our three-generation reproductive toxicity study of dietary BPA in CD(SD) rats (under OPPTS guideline 870.03800; U.S. EPA 1998) included six BPA dose groups (30/sex/group/generation) at 0.015–7,500 ppm (~ 0.001–500 mg/kg/day) (Tyl et al. 2002). We found no nonmonotonic dose–response patterns or effects on any parameters in the low-dose region (1 μg/kg/day to 5 mg/kg/day). We reported reduced adult body and organ weights at ≥ 50 mg/kg/day and renal and hepatic histopathology at 500 mg/kg/day. Reproductive/developmental toxicity was present only at 500 mg/kg/day in the presence of overt adult systemic toxicity. At doses < 500 mg/kg/day, we found no BPA effects on prostate weights (lobe or whole gland), acquisition of puberty, or other reproductive organ weights or histopathology in adult offspring. The systemic no observable adverse effect level (NOAEL) was 5 mg/kg/day, and the reproductive/developmental NOAEL was 50 mg/kg/day. BPA was not considered a rat selective reproductive/developmental toxicant.

Ema et al. (2001), funded by the Japanese Ministry of Health and Welfare, published a two-generation BPA rat study, with daily gavage dosages of 0 and 0.2–200 μg/kg/day under U.S. EPA GLPs and OPPTS TG (U.S. EPA 1998), adding endocrine-sensitive and neurobehavioral end points. They found no significant, compound-related effects in any parameter at any dose, consistent with our conclusions.

Myers et al. (2009) criticized our rat study for the absence of a positive control, although we reported successful use of 2.5 ppm dietary E_2_ (~ 180–200 μg/kg/day) in another dietary multigenerational study using the same rat source (Tyl et al. 2006). Biegel et al. (1998), using a 90-day plus one-generation study design, evaluated dietary E_2_ in CD(SD) rats; determined that dietary concentrations of 10 and 50 ppm were incompatible with pregnancy or offspring, and found the same effects at 2.5 ppm as we did. A “positive control” group is neither required nor routinely employed in guideline-compliant studies, because the study design and end points are validated (i.e., known to result in adverse consequences from an appropriate chemical by appropriate routes and doses). Neurotoxicity guideline protocols require annual evaluation of the test system with positive controls.

Because rats were cited as insensitive (or less sensitive than mice) to estrogens, mouse BPA reproductive toxicity studies were requested by European regulatory agencies. Our laboratory performed one- and two-generation reproductive toxicity studies of dietary E_2_ in CD-1 mice (Tyl et al. 2008a, 2008b) under OECD guidelines and GLPs, to establish appropriate end points and effects at low to high doses of an endogenous estrogen and to determine the appropriate dietary E_2_ positive control concentration/dose for the subsequent dietary BPA study in CD-1 mice. We evaluated dietary E_2_ concentrations from 0.001 to 50 ppm (~ 0.2 μg/kg/day to ~ 8 mg/kg/day). Doses ≥ 400 μg/kg/day (≥ 2.5 ppm) resulted in normal F_0_ mating behavior but no pregnancies or F_1_ offspring (Tyl et al. 2008a). Doses of ~ 0.2–80 μg/kg/day E_2_ (0.001–0.5 ppm) resulted in appropriate reproductive functions over one and two adult/offspring generations (Tyl et al. 2008a, 2008b), with anticipated dose-related effects on acquisition of puberty [accelerated vaginal patency (VP), delayed preputial separation] and AGD (shortened in males); these doses also affected weights of ovaries, uterus, cervix, and vagina, but not prostate and nonmonotonic dose–response curves for any parameter. The most complete spectrum of E_2_-dependent effects occurred at 0.5 ppm in the E_2_ two-generation study (Tyl et al. 2008b), such that E_2_ concentration (~ 80–120 μg/kg/day) was the positive control for the mouse dietary BPA study.

The mouse study (Tyl et al. 2008c), under OECD GLPs and guidelines, involved two vehicle control groups (0 ppm), six dietary BPA dose groups (0.018–3,500 ppm; 0.003–600 mg/kg/day), and one positive control group (E_2_), with 28 mice/sex/group/generation over two parental and off-spring generations. One additional postwean F_1_ male/litter was retained through adult necropsy and histopathology. E_2_ results confirmed sensitivity of CD-1 mice to estrogen. We found no BPA-related effects on mating, fertility, or gestational indices or on ovarian primordial follicle counts, estrous cyclicity, precoital interval, neonatal offspring AGD, sex ratios, survival, andrology, reproductive organ weights, or histopathology (including testes and prostate). Adult systemic effects at 300 ppm (~ 50 mg/kg/day) included only liver histopathology; at 3,500 ppm (~ 600 mg/kg/day), there were reduced body weights, increased renal and liver weights, and histopathology in adult males, and transient effects on weanling body and organ weights, but no adverse effects on adult reproductive structures or functions. These transient effects were considered secondary to systemic toxicity. At lower doses (0.018–30 ppm; ~ 0.003–5 mg/kg/day), there were no treatment-related effects or nonmonotonic dose–response curves for any parameter at any dose. The systemic NOAEL was 30 ppm BPA (~ 5 mg/kg/day), and the reproductive/developmental NOAEL was 300 ppm (~ 50 mg/kg/day). BPA was not considered a selective mouse reproductive or developmental toxicant (Tyl et al. 2008c). We identified the same BPA systemic and reproductive/developmental NOAELs (and sensitivity comparable to similar dietary E_2_ intakes) in rats and mice, with no BPA effects on the prostate weight or histopathology. Strain differences in response to estrogens in rats (and mice) vary across tissues, so no strain can be considered more sensitive than another (Howdeshell et al. 2008). E_2_ activities via estrogen receptor-α in the reproductive tract did not display major strain differences in OECD multilaboratory rat uterotrophic assay validation studies; oral BPA was only a weak partial agonist at 400–600 mg/kg/day (Kanno et al. 2003).

## Pharmacokinetics of BPA

The lack of low-dose effects in oral studies is consistent with BPA pharmacokinetics. Absorption, distribution, metabolism, and elimination (ADME) and any subsequent BPA toxicity depend on route of administration. Oral BPA at low (or high) doses is almost completely (> 95%) monoglucuronidated by the intestine and/or liver before it enters general circulation (Inoue et al. 2001, 2003; Pottenger et al. 2000). BPA monoglucuronide (BPA-G) is inactive as an estrogen (Matthews et al. 2001; Snyder et al. 2000) and is rapidly and almost completely excreted by urine and feces in rats (Pottenger et al. 2000) and by urine in humans (Dekant and Völkel 2008; Völkel et al. 2002), with a half-life of approximately 4 hr. Although neonatal rats and human infants have less glucuronidation enzymes than adults, Domoradzki et al. (2003) reported that ≥ 99% of oral 1 mg/kg BPA (333- to 1,000-fold higher than human perinatal or adult exposures) in neonatal rats was glucuronidated. Calafat et al. (2008) confirmed infant BPA metabolism, reporting that > 90% BPA in urine from premature babies was present as BPA-G. There is much higher (and longer) bioavailability of parent BPA from non-oral routes of administration (Pottenger et al. 2000).

## Exploratory Research BPA Studies

Exploratory research studies evaluate early, molecular, nonvalidated end points but rarely follow these initial responses to long-term adverse consequences. If the study is well conducted and reproducible and the interpretation is clear, it can be used to support risk assessment. vom Saal’s laboratory (Nagel et al. 1997) reported enlarged prostates in F_1_ adult CF-1 mouse offspring (six or seven males per group) from *in utero* exposure on gestational days (GDs) 11–17 to oral BPA at 2 and 20 μg/kg/day. These effects on offspring prostate weights (no histopathology was performed) triggered robust evaluations by Cagen et al. (1999) and Ashby et al. (1999), who could not replicate Nagel et al.’s prostate findings. The studies by Cagen et al. and Ashby et al. were criticized for no response to 0.2 μg/kg body weight/day diethylstilbestrol (DES) as a “positive control,” which was recommended to the authors by vom Saal; however, oral, low-dose DES prostate effects reported by vom Saal et al. (1997) have never been replicated. Other small mouse studies reported low-dose BPA effects on fetal prostate morphometry (Timms et al. 2005), acceleration of puberty (not on VP but timing of first estrus relative to VP; Howdeshell et al. 1999), altered mammary gland development (non-oral BPA exposure; Markey et al. 2005; Munoz-de-Toro et al. 2005), and altered mouse behavior (oral BPA; Ryan and Vandenbergh 2006). These findings, not replicated by robust studies in CF-1 mice (Cagen et al. 1999) or guideline-compliant studies in CD-1 mice (Tyl et al. 2008c), likely prompted the CERHR (2007) to indicate “some concern” for BPA’s neurobehavioral effects and “minimal concern” for accelerated puberty and prostate weights. The NTP prepared its own BPA report (NTP 2008), expressing “minimal concern” for effects on mammary gland development and accelerated puberty, and “some concern” for neurobehavioral and prostate gland effects.

## Responses to Specific Criticisms by Myers et al. (2009)

Myers et al. (2009) criticized the GLP-compliant studies for serious conceptual and methodologic flaws, failure to replicate, and methods not sensitive or “state of the art.” They argued that industry-sponsored GLP studies cannot detect low-dose effects and attacked the BPA studies in CD(SD) rats (Tyl et al. 2002) because the study used an insensitive species and strain [although the NTP conducted low-dose, multigeneration reproduction studies with ethinyl estradiol (EE) and genistein in the SD rat and detected estrogenic effects (Latendresse et al. 2009)] and in mice (Tyl et al. 2008c) as “so flawed as to be useless” (Myers et al. 2009). Their specific criticisms and my responses are as follows:

### Claim 1

Myers et al. (2009) claimed that the positive control group used a very high E_2_ dosage (0.5 ppm in diet at ~ 0.080 mg/kg/day) in contrast to the mouse literature for E_2_ (e.g., Richter et al. 2007; vom Saal et al. 1997). They noted that the EFSA report (EFSA 2006) failed to acknowledge that only a very high positive control dose was sufficient to elicit effects, and therefore the studies by Tyl and colleagues were insensitive to any estrogen and thus inappropriate for use in a study to examine low-dose estrogenic effects.

### Tyl response

The dietary positive control (0.5 ppm E_2_) was selected based on one-generation (Tyl et al. 2008a) and two-generation (Tyl et al. 2008b) CD-1 mouse studies using E_2_ doses of 0.001–50 ppm (8 mg/kg/day), with the most complete spectrum of effects seen at 0.5 ppm [estrogenic effects were observed at 0.05 ppm (increased weanling uterine weight) with a NOAEL of ~ 1 μg/kg/day]. Because E_2_ was dietary (animals were fed *ad libitum*, with ADME occurring as they fed), it took higher feed doses versus gavage or other routes to achieve the same test chemical response. Because almost all human BPA exposure is oral (during episodic eating/drinking), dosed feed is the most relevant exposure route.

No studies mentioned by Myers et al. (2009) investigated E_2_ after oral administration: Gupta (2000), Timms et al. (2005), and vom Saal et al. (1997) used DES; Putz et al. (2001a, 2001b) used oral EE; Timms et al. (2005) used estradiol benzoate by subcutaneous injection in rats, and Leranth et al. (2008) used it by subcutaneous capsules in primates; vom Saal et al. (1997) used sub-cutaneously implanted Silastic E_2_ capsules; and Richter et al. (2007) used E_2_ (and BPA) in cell culture. We rejected EE (synthetic and greater oral bioavailability) and DES (multiple modes of action, no reproducible data that it is orally active at low doses) as positive controls.

In Table 9 of our paper (Tyl et al. 2008a), we summarized key findings in rats and mice at the highest dietary concentrations producing viable offspring (2.5 ppm in rats; 0.5 ppm in mice), at doses of similar order of magnitude: mice, ~ 0.08–0.12 mg/kg/day; rats, ~ 0.17–0.2 mg/kg/day. Although there are differences in some end points, effects of exogenous estrogen in rats and mice are similar at similar doses. The concerns about our positive control and dietary concentration are unfounded.

### Claim 2

Myers et al. (2009) claimed that the “large” prostate weights suggest poor dissection technique, and that use of dissection, weight, and histopathology of the seminal vesicles plus coagulating glands (SVCG) together was “inappropriate.”

### Response

Prostate weights (lobe or whole gland) in all mammals evaluated to date have increased with age as males mature sexually (Sinowatz et al. 1996, for mice), as observed in our CD-1 mice. Mean control mouse prostate weights by lobe and age in our studies are presented in Table 1. Table 2 shows mean mouse whole-prostate weights of mice of various ages reported by others.

My laboratory has extensive experience in weighing rat and mouse prostate glands at various ages. In the Hershberger inter-laboratory validation study, my laboratory had some of the most precise prostate weight data and robust treatment-related effects of the 17 participating laboratories (according to one senior OECD/U.S. EPA reviewer; personal communication). We performed power calculations on our rat and mouse prostate data versus data of Nagel et al. (1997). Because of larger sample sizes and smaller coefficients of variation, our data have greater power to detect small effects than the Nagel data (unpublished data). Examination of paraffin block faces and slides of the rat and mouse prostates in the our studies (Tyl et al. 2002, 2008c) indicated no evidence of extraneous tissue/fat or excessive inflammation.

vom Saal’s CF-1 offspring mice are routinely group housed by sex for several months beginning at weaning, and then singly housed for 1 month before necropsy. It is highly unlikely that 1 month of single housing compensated for months of group housing and its effect on male mouse sexual development; the dominant cage male develops large androgen-dependent accessory sex organs (ASOs), and subservient cage males have smaller ASOs (Bartos and Brain 1993). vom Saal’s CF-1 mice did not exhibit increased prostate weights with age, likely because of his postwean caging regimen. Adult control prostate weights in our reproductive toxicity studies with E_2_ (Tyl et al. 2008a, 2008b) and BPA (Tyl et al. 2008c) (Table 1) are well within the weight range of other published studies (Table 2), and, as expected, our mouse prostate weights increased with increasing age.

We dissected, weighed, and examined the SVCG together to prevent tissue damage from necropsy separation of these intimately associated organs for histopathology, especially in mice.

### Claim 3

Myers et al. (2009) and vom Saal (at an FDA hearing on BPA safety assessment held 16 September 2008 in Washington, DC) stated that our animals must have had high incidence and severity of prostatitis to account for increased prostate weights.

### Response

For male mice exposed to E_2_ (0, 0.001, 0.005, 0.05, 0.15, and 0.5 ppm), histopathologic prostate examination by an independent pathologist indicated incidences of chronic inflammation (plus mononuclear cell infiltration): for F_0_ males, 0.0%, 16.7%, 0.0%, 8.3%, 9.1%, and 0.7%, respectively (*n* = 11–25/group); and for F_1_ males, 4.0%, 9.1%, 9.1%, 0.0%, 15.4%, and 0.0% (*n* = 10–25/group) (Tyl et al. 2008b). For the mouse BPA study (Tyl et al. 2008c), F_0_ male incidences were 6.7%, 2.2% (vehicle controls), 5.6%, 0.0%, 0.0%, 5.0%, 0.0%, 0.0% (BPA low to high doses: 0.018, 0.18, 1.8, 30, 300, and 3,500 ppm BPA, respectively), and 0.0% (positive control group; 0.5 ppm E_2_), *n* = 45–10/group; for F_1_ males, 3.6% and 0.0% (vehicle control), 0.0%, 4.2%, 10.0%, 0.0%, 0.0%, 7.7% (BPA low to high doses), and 3.3% (positive control), *n* = 20–56/group. Histopathologic characteristics of spontaneous prostate lesions in CD-1 mice (Chandra and Frith 1992; Karbe 1987; Sugimura et al. 1994; Waymouth et al. 1983) include the following:

- Various degrees of inflammatory cell infiltration in interstitium
- Neutrophilic infiltration and cellular debris accumulation in acini
- Minimal reactive epithelial hyperplasia in involved acini (Suwa et al. 2002).

Our incidences in CD-1 mice match low incidences of prostatic inflammation (~ 4–5%) in many mouse strains, with no treatment- or dose-related changes in incidence or severity.

In our BPA and E_2_ studies (Tyl et al 2002, 2008a, 2008b, 2008c), experienced prosectors (initially trained by veterinary pathologists) dissected reproductive tract organs, including prostates for adult and weanling males. Technicians were blind for doses (i.e., doses designated by Rx number and color code) to preclude inadvertent bias during all evaluations. Necropsy order was randomized such that prosectors dissected animals from all groups on all days, when possible.

### Claim 4

Myers et al. (2009) claimed that the use of Purina Certified Ground Rodent Chow No. 5002 was inappropriate because Thigpen et al. (2003) had reported that this diet was high in phytoestrogens and interfered with exogenous estrogen activity.

### Response

Most exploratory research studies, including those of vom Saal and colleagues, do not report phytoestrogen content of their diets, so it is difficult to compare diets used across laboratories. In our studies, mean phytoestrogen content of Purina 5002 feed batches was 192 ppm genistein, 177 ppm daidzein, and 45 ppm glycitein for mice (Tyl et al. 2008c), and 128 ppm genistein, 131 ppm daidzein, and 50 ppm glycitein for rats (Tyl et al. 2002). These levels were relatively consistent across batches within studies and across time.

Thigpen et al. (2003) investigated acquisition of VP in CD-1 mice with various diets of low to high phytoestrogen content [specifically daidzein and genistein (D&G)] and concluded that higher dietary phytoestrogens accelerated VP.

Our mouse data indicated control F_1_ mean ages at VP were 27.0 (Tyl et al. 2008b) and 29.4 (Tyl et al. 2008a) days, with no phytoestrogen effect, because Thigpen et al. (2003) reported the following percentages of females acquiring VP at 22–30 days of age: for low D&G diets (0–20 μg/g), 3.6–86.6%; for midrange D&G diets (101–210 μg/g), 23.8–87.7%; and for high D&G diets (270–370 μg/g), 37.5–96.0%. Sensitivity of VP to exogenous estrogen was demonstrated in our E_2_ mouse studies, because F_1_ VP age was significantly accelerated to 21.3 days by 0.5 ppm (Tyl et al. 2008a), to 21.0 days by 0.15 ppm, and to 20.7 days by 0.5 ppm (Tyl et al. 2008b). In our mouse BPA study (Tyl et al. 2008c), F_1_ VP was accelerated to 20.6 days in the 0.5-ppm E_2_ positive control group, compared with 25.5 days in controls. Our feed did not mask endocrine-sensitive responses to E_2_ (BPA doses administered were up to 7,000× higher).

### Claim 5

Myers et al. (2009) questioned our use of CD-1 Swiss mice from Charles River Laboratories (citing strain and source differences as the basis for differing results).

### Response

We purchased CD-1 mice from Charles River Laboratories (Raleigh, NC) for each study. We do not maintain in-house colonies because closed colonies (such as the CF-1 mouse colony maintained in vom Saal’s laboratory since 1979) typically suffer from founder effects and/or genetic drift over time; vom Saal recently terminated his CF-1 mouse colony and began using CD-1 mice. Charles River International Gold Standard CD-1 mice are maintained by matings across their breeding facilities to preclude/minimize such effects.

### Claim 6

Myers et al. (2009) criticized our use of gross examinations, organ wet weights, tissue histopathology, and systemic and reproductive/developmental landmarks because they “were established procedures by the 1950s.”

### Response

These parameters have been used for many years because they are sensitive, relevant, and validated. Newer end points have been added as they are validated (see above).

### Claim 7

Our reproductive toxicity study did not look at neurobehavioral end points.

### Response

Multigeneration studies are not designed to investigate neurobehavioral end points, although daily clinical observations in our studies (Tyl et al. 2002, 2008c) gave no indications of neurotoxicity/neurobehavioral effects after BPA exposure (e.g., normal mating behaviors, nest building, nursing, pup retrieval, offspring landmark acquisition). Ema et al. (2001) included standard neurobehavioral end points and observed no effects in their two-generation, low-dose (0.2–200 μg/kg/day) BPA study. Specific U.S. EPA and OECD guidelines exist for adult and developmental neurotoxicity studies; definitive BPA investigations using these guidelines are planned or under way by the FDA and industry.

### Claim 8

Myers et al. (2009) said that the large number of animals overpowered the study and violated National Institutes of Health (NIH) and federal guidelines.

### Response

Guideline studies are performed not just to detect adverse effects but also to ascertain dose–response relationships and to provide some assurance of safety. These studies therefore require different statistical considerations and experimental power. OECD and U.S. EPA reproductive toxicity guidelines require ≥ 20 pregnant females at term per group. Because mice are not typically used in guideline-compliant reproductive toxicity studies, and we evaluated ordinal (as well as continuous) data, we used 28 mice/sex/group/generation. Studies with small groups and group sizes are of limited utility, and inappropriate statistical analyses, sometimes seen in exploratory studies (e.g., CERHR 2007), cannot determine whether there are treatment-related effects.

Larger numbers of animals per group provide more statistical power to detect intergroup differences. A dose–response trend suggests treatment-related effects, whereas no dose–response trend implies that effects might not be treatment related, so studies with only one or two dose groups are of limited utility.

### Claim 9

Myers et al. (2009) listed BPA low-dose effects not present in our GLP studies, including altered metabolism (e.g., Alonso-Magdalena et al. 2006), adiponectin secretion (Hugo et al. 2008), epigenetic programming causing increased susceptibility to prostate cancer (Ho et al. 2006), changed gene expression and morphology of mammary glands (Durando et al. 2007; Munoz-de-Toro et al. 2005; Soto et al. 2008), neuroanatomic (Leranth et al. 2008) and neurobehavioral effects (Ryan and Vandenbergh 2006), and altered offspring reproductive systems (Markey et al. 2005; Newbold et al. 2007).

### Response

Low-dose BPA effects are from nonvalidated end points in exploratory studies, most after nonrelevant routes of administration: Soto et al. (2008), Markey et al. (2005), Munoz-de-Toro (2005), and Durando et al. (2007) used subcutaneous osmotic mini-pumps; Newbold et al. (2007) and Ho (2006) injected BPA; and Leranth et al. (2008) implanted subcutaneous BPA capsules. Guideline-compliant studies must use appropriate routes and validated end points to detect adverse outcomes, for example, changes in survival, growth and/or development, body and/or organ weights, histopathology, and systemic and reproductive organ functions.

### Claim 10

Myers et al. (2009) argued that “NIH-funded research [is] subject to more stringent reviews than GLP.” Their argument was that NIH applications require evidence of principal investigator competence, state-of-the-art methods, equipment, laboratory environment, publication of their findings in peer-reviewed journals, and are challenged by independent efforts to replicate their findings.

### Response

Risk-relevant guideline studies (including our BPA work) are not exploratory or “basic” research studies; they do not include unvalidated, cutting-edge techniques because they are required to use validated end points and parameters; they are welcomed, reviewed by experts, and published in highly respected journals.

A former *Nature* editor (Jennings 2006) stated that “scientists understand that peer review per se provides only a minimal assurance of quality, and that the public conception of peer review as a stamp of authentication is far from the truth.”

GLP-compliant studies require that all participants have current training files; have documentation showing that relevant SOPs are read, understood, and used; and are under independent quality assurance oversight. GLPs require that all data be included in the study report (and retained). Commonly, negative data are not published by many exploratory/basic research studies from universities or NGOs because they are not considered critical, whereas all data (positive/negative) are retained in guideline studies and are important in weight-of-evidence evaluations. Our mouse BPA study (Tyl et al. 2008c) was also under formal oversight by noted reproductive toxicologists from the United Kingdom, Denmark, Germany, the Netherlands, Sweden, and the European Chemicals Bureau.

Current publication environments, which may distort science, including limited journals with high impact, limited journal space, bias toward exciting positive studies and certain authors, and so forth, concern the National Institute of Environmental Health Sciences and academia (Young et al. 2008).

There is no guarantee that basic research, federally (or other) funded and published in peer-reviewed journals, is reproducible or subject to more stringent review. The following are specific examples of nonreproducible data:

- Sharpe et al. (1995) reported that gestational and lactational exposure of rats to xenoestrogens resulted in reduced testicular size and sperm production in adult offspring, but they rescinded their findings (Sharpe et al. 1998) because neither they nor other laboratories could replicate their findings.
- vom Saal’s laboratory (Nagel et al. 1997) reported significantly enlarged prostates and significantly decreased epididymal sperm counts in F_1_ adult CF-1 offspring from maternal BPA oral gavage at 2 and 20 μg/kg/day on GDs 11–17 (the sperm findings were not confirmed at the NTP low-dose workshop, held at Research Triangle Park, NC, in 2002). Robust attempts to replicate vom Saal’s prostate findings by Cagen et al. (1999) and Ashby et al. (1999) were unsuccessful (discussed above). Although not a direct comparison with work by Nagel et al. (1997) in CF-1 mice, Howdeshell et al. (2008) reported that EE gavage dosing of Long-Evans rat dams at 50 μg/kg/day from GD7 to post-natal day (PND) 18 reduced offspring body and androgen-dependent organ weights. However, BPA gavage dosing at 2, 20, or 200 μg/kg/day from GD7 to PND18 did not “significantly affect any male endpoint, including no effects on androgen-dependent organ weights or epididymal sperm count” (Howdeshell et al. 2008).
- Hunt et al. (2003) reported that BPA from cracked polycarbonate caging caused meiotic aneuploidy in genetically susceptible female mice, and dosing this strain with 20 μg/kg/day BPA for 7 days caused chromosome congression failure (disturbed metaphase chromosome alignment) during oocyte meiosis. A repeat of Hunt et al.’s study by Eichenlaub-Ritter et al. (2008) found no BPA-induced aneuploidy. Pacchierotti et al. (2008) found no hyperploidy/polyploidy in oocytes/zygotes (but reported increased meta-phase II oocytes with prematurely separated chromatids after chronic BPA exposure).
- Anway et al. (2005) reported that maternal intraperitoneal injection of vinclozolin (100 mg/kg/day) during early gonadal differentiation (GDs 8–15) in rats induced epigenetic alterations, which caused testicular histopathology, reduced sperm counts, and tumors in four offspring generations. Subsequent studies could not replicate these findings using intraperitoneal injection (Shirai et al. 2006; Strauss et al. 2009) or oral dosing (Schneider et al. 2008), and Anway et al. (2008) could not reproduce their original findings. Gray and Furr (2008) gavaged pregnant SD rats with 100 mg/kg/day vinclozolin on GDs 8–15 or on GDs 13–17 during androgen-dependent sex differentiation (controls dosed on GDs 8–17). These authors found no effects on F_1_ male AGD, retained nipples/areolae, hypospadias, or fertility after GD 8–15 exposure, but they did find statistically and biologically significant effects on these parameters after GD 13–17 exposure, as expected (vinclozolin is an androgen-receptor antagonist). Normal testis histopathology and epididymal sperm counts in F_2_/F_3_ generations indicated that vinclozolin-related effects (from exposed F_0_ dams) were not transmitted to unexposed F_2_ and F_3_ offspring (Furr and Gray 2009).

There are also cases of data manipulation/fraud in NIH-supported, peer-reviewed research—more than 30 cases documented in the past 3 years according to the U.S. Public Health Service’s Office of Research Integrity (2009). One example is the study of Arnold et al. (1996), in which the authors reported huge synergistic effects of endocrine disruptors in the yeast estrogen assay *in vitro*. McLachlan (1997) rescinded that paper because neither his laboratory nor others could replicate the findings. It was later determined that there was scientific misconduct and the original data were fabricated (NIH 2001).

Bell (2008) stated that “a failure in replicability has blighted much of the low-dose BPA literature and it is essential to determine which, if any, of these findings are capable of independent replication.” Ioannidis (2005) provocatively stated that “most published research findings are false,” because the smaller studies have less power and therefore more bias, so the effects reported are less likely to be true.

### Claim 11

Myers et al. (2009) stated that GLP specifies nothing about the quality of the research design, the skills of the technicians, the sensitivity of the assays, or whether the methods employed are current or out of date. (All of the above are central issues in the view of a grant proposal by an NIH panel.)

### Response

GLP regulations are the only international legal framework that establishes quality standards and independent government-mandated inspections of facilities, study data, and staff training, with severe monetary/legal sanctions for noncompliance (see, e.g., Office of Research Integrity 2009). Many academic institutions are implementing GLPs up to full compliance, including quality assurance oversight. Unlike basic research, GLPs require documented ownership of data (regardless of collection media), verification of valid study design, protocol amendments for planned changes, protocol deviations for unplanned changes, retention of all data, and appropriate statistical analyses to ensure confidence in the study design, performance, and conclusions, providing assured sensitivity, reliability, and validity critical to formal risk assessment.

Designs of BPA guideline-compliant, reproductive toxicity studies (e.g., Ema et al. 2001; Tyl et al. 2002, 2008c) and other comprehensive studies (e.g., Ashby et al. 1999; Cagen et al. 1999; Howdeshell et al. 2008) include adverse end points that would result from effects, and in some cases include effects themselves, reported by the small, exploratory research studies. The striking conclusion is that none of these guideline-compliant or comprehensive studies, regardless of sponsorship, has been able to replicate effects reported for low-dose BPA on prostate or other male/female reproductive structures or functions. The novel, nonvalidated end points evaluated by small, basic research studies were not evaluated in the guideline-compliant studies, but any adverse consequences from them, as required for risk assessment, were sought and not found.

## Weight of Evidence

During weight-of-evidence evaluations, experts evaluate relevant articles and reports, with certain study designs and/or end points assigned greater/lesser weight. A number of BPA weight-of-evidence assessments (e.g., EFSA 2006; Goodman et al. 2006, 2008a, 2008b; Gray et al. 2004; Willhite et al. 2008) concur that studies do not support the hypothesis that low-dose oral BPA adversely affects human reproductive/developmental health.

## Conclusions

*Ad hominem* attacks are not appropriate or helpful in discussions of scientific and regulatory concerns. This controversy can be resolved only if the debate returns to a professional level (Sagan 1996). To include new end points for hazard/risk assessment, we must validate the end points through rigorous regulatory acceptance processes, and replicate basic study effects using guideline studies. Success in validating relevant new end points, as well as performance of studies to follow early changes to subsequent adverse consequences *in vivo*, may provide “phenotypic anchoring” needed to use such end points for future hazard evaluations and risk assessments.

## Correction

In Table 1 of the original manuscript published online, the age of F_1_ animals at termination was given as 16 weeks. The animals actually ranged in age from 16 to 19 weeks, based on time during the cohabitation period that mothers were inseminated and when pups were delivered [Tyl RW. Ages of mice in the two-generation BPA study (Letter). Toxicol Sci (in press)]. The table has been corrected here.

## Figures and Tables

**Table 1:** CD-1 (Swiss) mouse control male prostate weights (mean ± SD).

Reference	Age (weeks)	Terminal body weight (g)	Mean absolute prostate weight (mg)		
			Whole prostate	Ventral lobe	Dorsolateral lobe
Tyl et al. 2008a^a^	12–13 (F_0_)	41.19 ± 1.08	43.2 ± 4.7	ND	ND
Tyl et al. 2008b^b^	19 (F_0_)	36.66 ± 0.77	82.0 ± 6.0	39.0 ± 4.0	42.0 ± 4.0
	16–19 (F_1_)	37.37 ± 0.71	58.0 ± 3.0	25.0 ± 2.0	34.0 ± 2.0
	18 (F_1_ retained)	38.89 ± 1.12	58.0 ± 5.0	24.0 ± 2.0	34.0 ± 4.0
Tyl et al. 2008c^c^	19 (F_0_)	39.53 ± 0.50	72.5 ± 3.6	34.8 ± 2.6	37.7 ± 1.7
	16–19 (F_1_)	38.89 ± 0.53	71.9 ± 2.2	26.5 ± 1.4	45.8 ± 1.7
	18 (F_1_ retained)	40.46 ± 0.67	74.4 ± 2.9	28.8 ± 2.4	45.6 ± 2.2

ND, not determined.

a Ten F_0_ males in control group.

b Twenty-five males in control group/generation.

c Fifty-six males in combined control groups/generation.

**Table 2:** Prostate weights in adult male control mice.

Reference	Mouse strain	Age (weeks)	Whole prostate weight (mg)
Ashby et al. 1999^a^	CF-1	25–26	~ 50
Cagen et al. 1999^a^	CF-1	13	~ 40
Gupta 2000	CD-1	8.5–9	~ 40
Heindel et al. 1995	CD-1	16–17	~ 45.9
Morrissey et al. 1988	CD-1	23	58.3
Nagel et al. 1997^b^	CF-1	27–28	41
Nonneman et al. 1992^b^	CF-1	14–15	49–59
Ruhlen et al. 2008	CD-1	13	~ 42
Takahashi and Oishi 2006	CD-1	8.5–9	16–34
vom Saal et al. 1994^b^	CF-1	15	50
vom Saal et al. 1997^b^	CF-1	34–35	42
vom Saal et al. 1994^b^	CF-1	77	105

a Cagen et al. (1999) and Ashby et al. (1999) used CF-1 mice purchased from commercial suppliers.

b The closed CF-1 mouse colony (maintained at the University of Missouri–Columbia since 1979) has since been terminated by vom Saal.
